# Decision-Making During Asynchronous Electives: Insights From Emergency Medicine-Bound Medical Students

**DOI:** 10.7759/cureus.60784

**Published:** 2024-05-21

**Authors:** Aarti Jain, Michael Shamoon, David Diller, Jeffrey Riddell

**Affiliations:** 1 Emergency Medicine, Keck School of Medicine of University of Southern California, Los Angeles, USA

**Keywords:** blended learning, motivation, distance learning, undergraduate medical education, asynchronous

## Abstract

Introduction

While asynchronous learning is gaining popularity, little is known about learners’ decisions regarding compliance with assigned asynchronous material. We sought to explore how medical students make decisions about the use of their time when engaging in asynchronous learning during the residency interview season.

Methods

After implementing a four-week blended elective for emergency medicine-bound fourth-year medical students, we conducted a mixed methods study with an explanatory sequential design. We analyzed weekly surveys regarding accountability and barriers to assignment completion and conducted semi-structured focus groups exploring the decisions students made regarding compliance with asynchronous assignments. Using a constructivist approach, we performed a thematic analysis of the transcripts.

Results

The average assignment completion rate was 36%, with the highest rates for podcasts (58%) and the lowest rates for textbook readings (20%). Compliance with assignments was enhanced by a desire for increased ownership of learning but was hindered by a lack of accountability, learner burnout, and higher prioritization of interviews. Students preferentially selected resources that were shorter in length, entertaining, and more convenient for travel.

Conclusion

Our study highlights factors impacting student compliance when engaging in asynchronous learning and offers insights into educational and institutional strategies that can be utilized to enhance learner motivation.

## Introduction

Given the increasing scheduling demands of the residency interview process [1,2], senior medical students report frustration with the lack of flexibility in medical school course offerings during the interview season [1,3,4]. Students often select “easier” electives, disrupt classes with prolonged and frequent absences, or simply opt not to schedule educational electives during heavy interview months [3,4]. In response, several medical schools have implemented “flexible electives” during the residency interview season, recognizing the potential for asynchronous or blended courses to overcome time and distance barriers to education [5,6]. 

While asynchronous learning is gaining popularity [7-10], studies assessing learning outcomes have demonstrated mixed results [11,12]. Specifically, there are concerns that compliance with assigned asynchronous material may be low [8,13]. Scholars have cautioned against “jumping on the asynchronous bandwagon,” specifically urging qualitative exploration to begin understanding the myriad of contextual factors associated with learning in asynchronous courses [9].

Existing studies of asynchronous courses have examined learner reactions and educational effectiveness but have neglected to consider how learners behave when engaging with asynchronous material [11,12]. Questions around the factors that influence learner prioritization of asynchronous assignments, barriers and incentives to compliance, and students’ preferred modalities for the delivery of asynchronous material remain unanswered. 

A deeper understanding of learner behavior is necessary if we are to reconcile inconsistencies in the reported effectiveness of asynchronous courses and create electives that provide maximal educational value for students during interview season. Therefore, we sought to describe the ways medical students make decisions about the use of their time during a distance elective, and how their decisions about compliance with asynchronous assignments might be impacted by the context of the residency interview season. 

## Materials and methods

This study utilized a fixed mixed methodology with a sequential explanatory design [14]. Quantitative data were collected and analyzed before the initiation of the qualitative phase and informed qualitative research questions, sampling, and data collection. The rationale for this approach is that the qualitative phase allowed for the corroboration of quantitative results through triangulation and enhanced complementarity, where the thematic analysis clarified, explored, and contextualized quantitative survey results in more depth [15]. 

Curriculum

During the peak residency interview months of November and December 2018, we implemented a four-week blended learning elective for emergency medicine-bound fourth-year medical students. A total of eighteen students enrolled in the course with nine students participating in each four-week elective block. Weekly course activities were organized around common chief complaints seen in the emergency department (ED). Each week, students engaged in four days of multimodal self-study and independent case preparation, with one day of on-campus progressive disclosure cases, simulation-based learning, and procedural training in a fresh tissue cadaver lab. For the self-study portion, students followed a detailed day-to-day schedule that included emergency medicine-specific resources in multiple formats (e.g. podcasts, textbooks, blog posts, online board review videos, and question banks). A more detailed description of the curriculum is available in the Appendices (Appendix 1).

Quantitative

We sent students anonymous online weekly surveys regarding accountability and barriers to completion of assignments during the course (Appendices (Appendix 2)). The surveys allowed us to collect information about behaviors that were not directly observable (e.g. completion of asynchronous assignments) and to measure psychological constructs that are challenging to quantify (e.g. attitudes and opinions about educational resources) [16]. The surveys were initially developed by two authors (AJ, MS) and were then assessed for clarity and relevance to the construct by the remaining two authors (DD, JR) [16]. They were also first pre-tested with individuals not involved in study authorship to evaluate response process validity [16]. Survey items asked students to indicate which of the assigned resources they had used in the past week, report barriers to the completion of assignments, rank the usefulness of the various modalities, and indicate the number of residency interviews they attended. Descriptive statistics were used to evaluate responses to scaled items. Differences in reported completion rates between modalities were analyzed with ANOVA using IBM SPSS Statistics for Windows, version 28 (IBM Corp., Armonk, N.Y., USA). Pearson correlation coefficient was used to examine the relationship between the number of interviews and assignments completed.

Qualitative

We drew from a constructivist approach and performed thematic analysis to explore how students made decisions about the use of their time during an asynchronous elective [17]. The authors acknowledge that our specific experiences and perspectives may have contributed to assumptions and preconceptions that shaped the research process [18]. The lead author (AJ) is a faculty member in emergency medicine who developed the curriculum for the asynchronous course and served as the primary educator for all on-campus sessions. Her collaborators include an assistant program director of an emergency medicine program who has conducted research on curricular evaluation (DD) as well as a medical education fellow (MS) in the Department of Emergency Medicine. The last author (JR) is a faculty member in emergency medicine with experience in qualitative medical education research, and whose scholarly focus involves educational technology. None of the authors participated in asynchronous courses in their medical school curricula, but all four authors completed asynchronous courses during Master's degree or other professional development programs. 

After obtaining informed written consent, in April 2019 we conducted two one-hour semi-structured focus groups with four course participants in each group. Voluntary participation was solicited via email invitation and each participant was provided with lunch for their time. Focus group sessions were facilitated by one of the authors (JR) and included predetermined, open-ended interview questions and probes intended to explore student experiences during the flexible elective as well the decisions they made regarding compliance with, and triage of, assigned asynchronous study material. Questions that were included in the focus group interview guide were informed by our quantitative data analysis and are available in the Appendices (Appendix 3). Focus group interviews were audio-recorded, transcribed, and de-identified prior to analysis. 

All four authors (AJ, MS, DD, JR) analyzed the transcripts in an iterative manner to identify patterns of meaning within the data and perform inductive thematic analysis [18]. Two authors (AJ, MS) initially analyzed both transcripts and developed open codes. All four authors (AJ, MS, DD, JR) then refined code definitions, applied them to both transcripts and reviewed the final coding structure to develop thematic categories. Generated themes were reorganized until consensus was achieved by the group. During the data analysis process, the authors utilized debriefing, memoing, and reflexive commentary as strategies for establishing trustworthiness [18]. 

## Results

Quantitative

**Figure 1:**
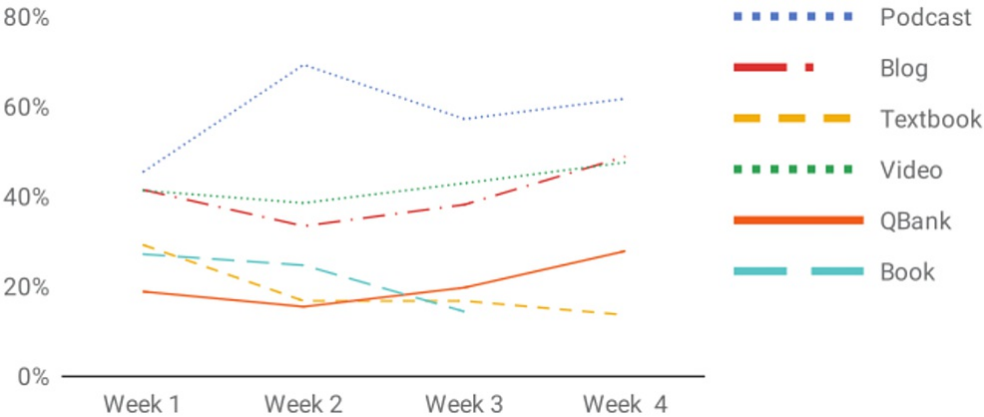
Assignment completion rates by modality

A total of 18 students participated in the blended course. Out of 72 possible surveys, they completed 56 (78%). Students reported completing an average of 36% of asynchronous assignments each week. The lowest percentage of assignments completed by a student in one week was 3%, while the highest completion percentage was 83%. When stratified across assignment modality, participants reported completing podcast assignments significantly more frequently than assigned textbook readings (F(1,27) = 9.66, p=.004), completing 58% of podcast assignments and only 20% of assigned textbook readings overall. Additionally, while completion of podcasts, blogs, videos and question banks increased over the four-week duration of the course, rates of completion for books and textbooks declined (Figure 1). On a 5-point ranked Likert scale, ranging from “Very Unhelpful” 1 to “Very Helpful” 5, participants rated the educational value of podcasts and blogs as an average of 4.6 and 4.4, respectively, whereas they rated textbook readings as 4.2 and question banks as 4.1. The number of interviews students attended and the percentage of assignments completed that week were weakly positively correlated (r(55) = 0.013, p = .93). When asked about barriers to completing assignments, respondents cited a “heavy interview / travel schedule'' 39% of the time and “life events” 18% of the time. When asked about how they prioritized which assignments to complete, participants reported modality as the primary reason 33% of the time and anticipated time required to complete an assignment 32% of the time.

Qualitative

Participants’ decisions about compliance with asynchronous assignments were made in the context of a shifting professional and learner identity. They described a tension between a desire for increased ownership of learning and a perceived lack of accountability for assignments. Compliance was hindered by learner burnout and by students prioritizing interviews over coursework. When triaging assigned asynchronous material, students preferentially selected resources that were shorter in length, entertaining, and more convenient for travel. In general, they gravitated towards podcasts and away from assigned textbook readings.

Shifting Professional Identity From Student to Physician

Study participants noted a shift in their professional identities during their final year of medical school, evolving from passive medical students to soon-to-be physicians craving agency in their educational trajectory. In this context, they expressed a clear preference for educational opportunities that would prepare them for their upcoming residency training and “help [them] with [their] career going forward.” (Focus Group (FG) 2) While didactic sessions from earlier years of medical school were described as “passive, sitting in the back of a dark room listening to people talk,” students now expressly sought the ability “to be a little more selfish and tailor [their] learning to things that [they’d] be using as a physician.” (FG1) Rather than feeling “distanced” from their learning and being expected to sacrifice their individual learning goals to “grind and be a team member,” they desired a “very involved and active” role in their education (FG1). When describing the purpose of the fourth year of medical school, one student stated: 

At this point in the year or at this point in medical education, for me I viewed this course and a lot of other clerkships as... It’s not so much just doing things to just complete them and getting the grade or getting through it. Now, I look at things, “What am I actually going to get from this...that I’m really going to learn and that’s going to help me in residency and beyond. (FG2)

In asserting this changing professional identity, they “tried to get rid of as much busy work as possible” and resented obligations that forced them to “twiddle [their] thumbs and check boxes” (FG1). They expressed a desire to be treated as “adults,” expressing little tolerance for educational activities that did not explicitly prepare them for their upcoming residency training.

Tensions

Autonomy vs. accountability: In the context of this shifting professional identity, students expressed a desire for ownership of their learning agendas. One course participant noted that the asynchronous format aligned with this objective, stating “We were learning for learning’s sake. What we chose to read, we chose to read” (FG1). While enjoying the opportunity to be “very in control of [their] learning”, (FG1) they acknowledged that the perceived absence of accountability in asynchronous learning decreased their motivation to comply with assignments. One student described the tension between these competing priorities: 

Because there were no consequences for not doing the at-home assignments, that did create a lack of motivation. We weren’t going to be tested on it. At the same time I really enjoyed that, because that’s the part, as learners, where I just want to be responsible for what I think is important for me. (FG1)

While on the one hand, students craved “assignment[s] you’re accountable for” in order to provide extrinsic motivation for compliance with asynchronous material, they also desired independence and trust that they would be intrinsically motivated by “thinking like a resident,” “being excited about going into this field,” and assignments “relevant to what I was focused on getting my future into” (FG2). One student stated, “When I’m really making decisions, I’m going to feel a lot more invested and a lot more excited about my learning” (FG1).

Desire to learn vs. burnout: Students’ desire for learning opportunities during the residency interview season was also threatened by burnout caused by the stressors of this time period. While expressing a desire for courses that were “win-win, where [they were] still learning but [were] able to go to interviews,” (FG2) they admitted that, at this point in the year, they had “no more emotional capacity to give to medicine” (FG1) Students described an accumulation of stressors, recalling that “from January to October, it was constantly being on rotations, sub-Is where you’re on all the time” and “then you’re on the interview trail and your fake smile and trying to put your best foot forward...all that stress eats away at you” (FG1). As a result of this increased stress, one student stated:

I kind of lost my joy for learning... I didn’t have anything left to give and I didn’t realize it until afterward now that I’m all excited about learning again. But my life was just automatic and I was just very tired. (FG1)

At the same time, students reported a desire for mental stimulation and continued education during the interview months. They acknowledged that they “[didn’t] just want to have a bunch of free time off” (FG2) and that “there were days where I didn’t have anything to do...and I would’ve just been bored out of my mind if I didn’t have something to do. So, [course assignments] would be an excuse to just grab a coffee and knock some stuff out for a couple hours” (FG1).

Desire to learn vs. desire to match: Students placed a higher priority on attending residency interviews than completing their asynchronous assignments. Despite “being in the middle of interview season and being excited about going into this field,” (FG2) students unequivocally stated that interviews were their priority. They noted that “our job fourth year is to match” (FG1) and the “objective of med school is to get into residency” (FG2) As such, when discussing the tension between complying with asynchronous assignments and focusing attention on residency interviews, one participant asserted, “That’s not a decision. It doesn’t matter. Our interviews come first no matter what” (FG1). Though students recognized that they “put [their] entire focus on interviews,” they acknowledged that this unidimensional approach was occasionally “all-consuming” and forced them to be “‘on’ all the time” (FG1). As a result, some students appeared to appreciate the distraction of asynchronous assignments stating, “It was something I could do quietly by myself and kind of recharge on the side and still feel like I was learning” (FG1) While justifying their higher prioritization of residency interviews, students acknowledged that their capacity to absorb and engage in educational content fluctuated during the interview season. Noting an inverse relationship between interview burden and desire for educational activities, one student recalled, “On the heavy [interview] weeks, the motivation was low. You were just trying to get through everything, like get through your life and interviews and stuff. But the weeks where you had less, you’d feel like you had more time and the motivation was a little bit higher” (FG1).

Triaging Asynchronous Material

Length of material: When prioritizing and triaging asynchronous assignments, students preferentially selected resources that were shorter in length. One student suggested that their decision to complete an assignment often depended on “how long it would take me to complete it. The readings I would cut out because that would take me the longest...that’s what I have as last priority because it’s going to take too long, it’s too hard, I’m not going to do it. (FG2). Students felt that shorter segments provided them with immediate gratification and a sense of accomplishment, stating that they “like to knock things off the checklist. It is satisfying to finish this, did that, check it off my box” (FG1). With lengthier assignments, they admitted that they would “just skip down to the bottom and hit the key points”, even employing the internet-famous gen-z descriptor “TLTR” (too long to read) (FG1).

Entertainment value: Students gravitated towards educational content with high entertainment value, stating that their “motivation was determined by...how much [they] enjoyed the material” (FG1). They reported a tendency to “dig into the [resources] that looked more entertaining”, stating that, when “they were cracking jokes in the articles...those I can breeze through” (FG1). In addition to recognizing the ease of engaging with entertaining educational material, they suggested that curated entertainment actually increased their retention of learning points. One student stated his appreciation for resources that “tell a story with their articles...I tend to enjoy them and learn something from them” (FG1). After identifying a particularly captivating resource, students would “look forward to those links” (FG1) and preferentially select them when triaging future weeks’ course materials. 

Convenience: Lastly, students prioritized assigned material that was more conducive to a busy travel schedule. They preferentially selected resources that were portable and allowed for multitasking, noting that they completed assignments “while walking to class” or “listened to them on the planes and while [they were] traveling and [they were] still learning” (FG2) They also appreciated the efficiency of portable asynchronous material, stating, “It was a really good use of time because it was really easy to put your headphones in when you’re waiting for your flight” (FG1).

General preference for podcasts and avoidance of textbooks: Overall, when selecting from assigned material in multiple modalities, students gravitated towards podcasts and avoided textbook readings. After initially attempting to comply with all assignments, one student noted, “I quickly realized what I was getting good bang-for-my-buck from and what I was sort of zoning out. And so, from there on...I would say ‘I’m going to listen to all the podcasts first...and I would generally save most of the readings for last” (FG2). Students admitted to poor compliance with reading material, reporting that it was too “dense”, (FG1) would “take too long,” (FG2) and was a “very low yield type of learning versus going through podcasts and videos” (FG2). Suggesting that textbook learning was “not going to be a valuable use of [their] time, [they] gravitated towards more of the online stuff, the podcasts” (FG1). They also appreciated the novelty of podcast-based learning in medical education, stating “that was a format I’m familiar with and appreciated”, but “was one of the first times in med school that I’ve had a podcast assigned” (FG2).

## Discussion

In this mixed methods study, we found that compliance with asynchronous assignments was poor and that students’ desire for greater autonomy and control of their learning agendas was countered by a perceived lack of accountability for assigned asynchronous materials as well as the heavy scheduling demands of the residency interview season. In the context of these time constraints and evolving professional identity, students favored shorter, more flexible, and more entertaining educational resources, particularly podcasts.

Our findings are consistent with prior studies suggesting that compliance during asynchronous medical courses may not match reported enthusiasm for asynchronous learning [7,13]. Even in their preferred educational modality of podcasts, course participants listened to only 58% of the assigned material. While other studies have reported poor compliance with asynchronous learning [7,13], there has been little exploration into the factors that might explain the discrepancy between learner enthusiasm and actual assignment completion rates. When discussing their motivations to comply with assignments, our study participants referenced a complex interplay between intrinsic factors, the learning environment, and external constraints, and suggested that, on any given day, motivation varied depending on the dynamic between these variables. These findings are in line with other studies that propose learner motivation to be multifaceted and sensitive to situational influences and argue against a dichotomous conceptualization of motivation as either intrinsic or extrinsic [19]. Given that external constraints such as the time burden of the residency interview season are often fixed, course developers and educators must focus their efforts on optimizing the learning environment and catalyzing intrinsic motivation in their learners. A pedagogical approach that allows learners to select educational content that fills self-identified educational gaps might increase compliance with assignments, as the presence of choices augments learners’ perceptions of competence and autonomy and often results in higher completion rates [20]. This may be particularly relevant for senior medical students who are evolving from “passive” students to increasingly autonomous learners. Offering course content in multiple modalities (e.g. textbooks, videos, podcasts) not only acknowledges individual learner preferences and provides opportunities for personalization, but can provide course directors with valuable insight into the appeal and effectiveness of specific course material. These increases in choices, however, must be balanced with sufficient parameters and instructor guidance to avoid overburdening students with the excessive decision-making required to “triage” all available options [21]. Learner autonomy may also be threatened by a lack of explicit connection between individual course assignments and stated learning objectives [22]. Clarifying the educational rationale of course assignments can improve autonomous motivation and behavioral engagement even during activities that may be perceived as “uninteresting” [23].

Regarding asynchronous learning in the context of the interview season, our findings are in line with other studies suggesting that fourth medical students perceive the primary purpose of the final year of medical school to be preparation for the match process and their future residency training [1]. Despite 80% of the blended course structure being flexible and asynchronous in nature, and course content aligned with students’ desires for residency preparation, students still suffered from low capacity to devote time and attention to both interviews and assigned educational content. This finding adds fuel to the argument that the residency interview process requires revamping and restructuring if educational motivation and value are to be preserved during these months [2]. In Canada, for example, all residency interviews are conducted during a designated two-week period, thereby minimized curricular disruptions [24]. Until the interview process is reformed, medical school expectations and policies about the expected number of hours of course content delivered during the interview season must be realistically decreased to accommodate the educational and time capacity of medical students during this time. Given that students cited heavy travel and interview schedules as the primary barriers to compliance with asynchronous assignments, future studies can examine whether compliance during flexible electives improves if the amount of total assigned coursework decreases. With the pandemic-induced shift towards virtual interviews, the travel burden of the residency interview should ostensibly be lessened and students may be able to devote more time and energy to electives held during interview months. If the virtual interview format persists, future studies should explore students’ compliance with asynchronous assignments in the absence of heavy travel and interview schedules. 

Podcasts are becoming increasingly utilized as tools in undergraduate medical education [25]. Our findings are consistent with previous studies suggesting that medical learners prefer podcasts to textbook learning [8, 26] and seek resources that provide entertainment value [27], are shorter [26, 27], and are conducive to multitasking [28]. In an era of increasing online educational resources and a growing number of educational podcasts, current trends suggest that podcasting will either informally or formally become integrated into undergraduate medical education [29]. While existing studies have characterized the scope of informal podcast usage in resident physicians [8,25], less is known about informal podcast adoption and usage patterns in medical students. If medical schools are to consider incorporating this learning modality into future asynchronous or blended courses [30], further information about the scope of, and motivations for, podcast usage among medical students would be valuable. It remains unclear whether medical students perceive podcasts to provide greater educational value, simply provide “edutainment,” or whether podcasts align with students’ shifting professional identities by increasing a sense of connection to their future professional communities [31]. Once informal podcast usage and preferences are better understood, medical schools can assess the urgency and best practices for the incorporation of podcast-based education into formal asynchronous and synchronous curricula. 

Limitations

The results of this study are limited by its small sample size and specialty-specific content. These findings may be representative of the contextual factors that are important to emergency medicine-bound individuals, but may not be generalizable to learners interested in other specialties. For example, blogs and podcast-based learning have gained more significant traction in the emergency medicine educational community than in other specialties [25]. Learner preferences for, or avoidance of, specific educational modalities may have been a consequence of the specific resources that were selected for this course as opposed to general feelings about the modalities themselves. Additionally, our reliance on self-reporting in the quantitative phase of data collection may have contributed to inaccurate assignment completion rates. 

This study is also subject to the inherent limitations of focus group dynamics. Participant candor about compliance issues may have been inhibited by the use of an emergency department faculty member as a focus group facilitator, however, given students’ open willingness to discuss lack of compliance with assignments, we suspect that the authenticity of their responses was mostly preserved. To limit students’ concerns that their responses would negatively impact their chances for a successful residency match, focus group interviews were conducted after match day. Additionally, focus group discussions may have been susceptible to a bandwagon effect, where learners concur with an opinion once it emerges, or social desirability bias, where diversity of opinions is suppressed.

## Conclusions

Our study explores how medical students make decisions about the use of their time when engaging in an asynchronous elective during the residency interview season and uncovers factors impacting compliance with asynchronous assignments. Given a self-described shift in the professional identities of learners, directors of asynchronous courses must aim to enhance motivation and compliance with assignments by augmenting learner perceptions of competence and autonomy and explicitly linking course activities to learning objectives. Medical institutions should also reconsider the academic expectations of learners during the residency interview season and proactively address the growing popularity of non-traditional learning modalities, such as educational podcasts. As asynchronous learning becomes more prevalent, future studies must continue to explore this discrepancy between enthusiasm for asynchronous learning and compliance with asynchronous assignments.

## Figures and Tables

**Figure 2:**
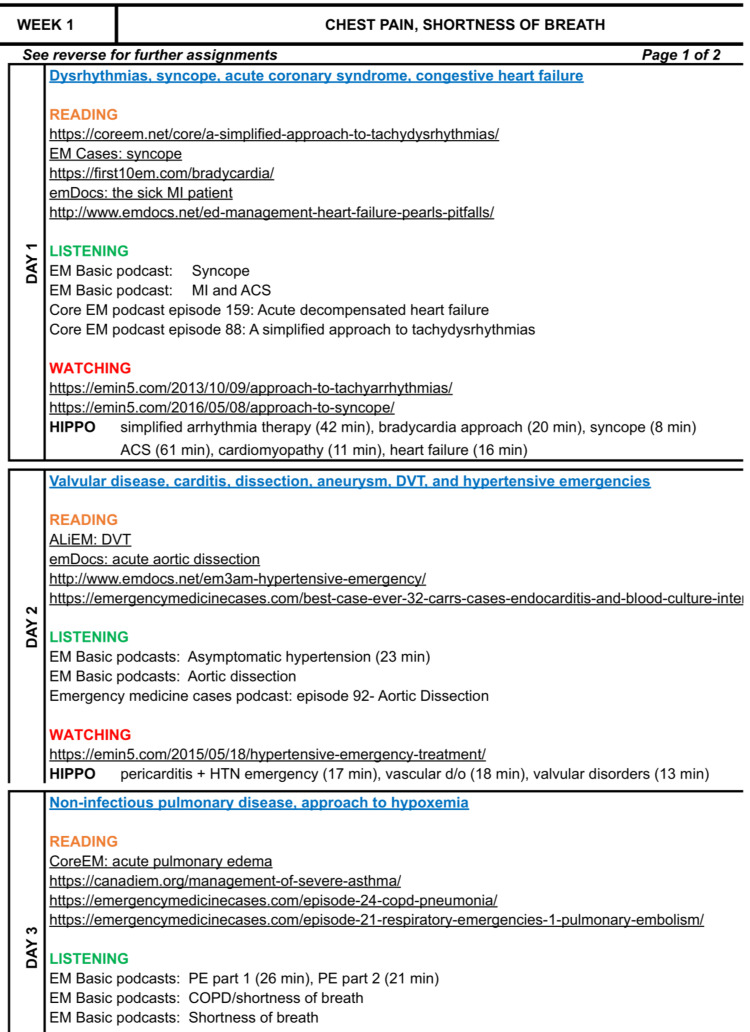
Appendix 1 Sample page from course curriculum.

**Figure 3:**
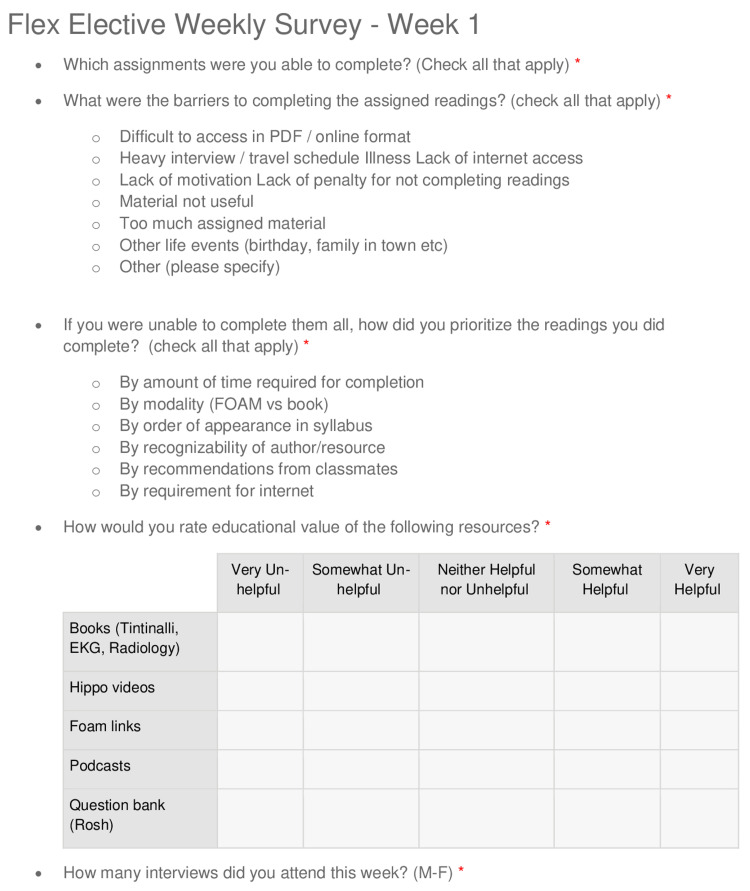
Appendix 2 Weekly online survey.

**Figure 4:**
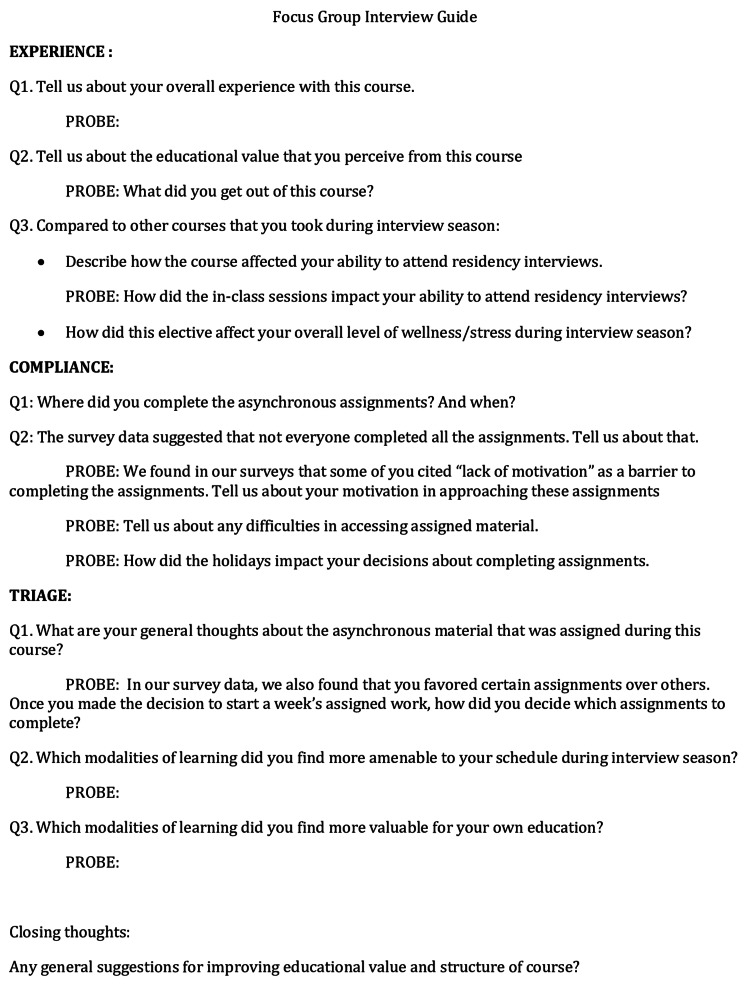
Appendix 3 Focus group interview guide.
